# Significance of Early Proton Beam Therapy Initiation in Achieving Complete Response in Pediatric Medulloblastoma: A Retrospective Study

**DOI:** 10.1002/cam4.71757

**Published:** 2026-03-31

**Authors:** Zhipeng Shen, Zhuo Hou, Wei Han, Yi Zhang, Weiling Zhang, Shuihua Wu, Jianfeng Liang, Chen Jiang, Zishen Wang, Wei Wang, Shuyan Zhang, Hideyuki Sakurai, Shosei Shimizu (Qingshui Xiangxing)

**Affiliations:** ^1^ Department of Neurosurgery, the Children's Hospital of Zhejiang University School of Medicine Zhejiang Hangzhou China; ^2^ National Clinical Research Center for Child Health Zhejiang Hangzhou China; ^3^ Institute of Engineering Tokyo University of Agriculture and Technology Koganei Tokyo Japan; ^4^ Department of Pediatric Oncology, Beijing Children's Hospital, Capital Medical University Beijing China; ^5^ Pediatric Internal Medicine, Beijing Tongren Hospital, Capital Medical University Beijing China; ^6^ Department of Neurosurgery, Hunan Children's Hospital Changsha Hunan China; ^7^ Department of Neurosurgery, Peking University International Hospital Beijing China; ^8^ Department of Neurosurgery, Affiliated Hospital of University of Science and Technology of China, Anhui Hefei China; ^9^ Department of Radiotherapy Physics and Technology, Hebei Yizhou Cancer Hospital Zhuozhou Hebei China; ^10^ Department of Radiology, Hebei Yizhou Cancer Hospital Zhuozhou Hebei China; ^11^ Department of Pediatric Radiation Therapy Center/Pediatric Proton Beam Therapy Center, Hebei Yizhou Cancer Hospital Zhuozhou Hebei China; ^12^ Department of Radiation Oncology, University of Tsukuba Tsukuba Ibaraki Japan

**Keywords:** complete response, initiation time, medulloblastoma, proton beam therapy, radiation therapy, tumor response

## Abstract

**Introduction:**

Proton beam therapy (PBT) has proven to be highly effective in treating pediatric medulloblastoma, offering both excellent therapeutic outcomes and reduced side effects. However, factors influencing tumor response following PBT remain poorly defined, including the optimal interval time between surgery and PBT initiation (ISP).

**Methods:**

This retrospective study analyzed data from 52 patients with postoperative residual medulloblastoma treated with PBT, focusing on the correlation between tumor response and ISP, as well as other variables associated with achieving complete response (CR).

**Results:**

The median follow‐up period was 12.5 months (5.3–20.3 months). Treatment response was assessable in all patients, with 26 (50.00%) patients in CR, 15 (28.85%) patients in partial response (PR), and 11 (21.15%) patients with stable disease (SD). Patients who initiated PBT within 31 days post‐surgery had a markedly higher CR rate (74.07%), while those with longer ISPs were more likely to exhibit PR (32.00%) or SD (44.00%). An improved tumor response was significantly associated with a shorter ISP (30.15 ± 5.31 days, *p* < 0.001). Univariate logistic regression identified Chang staging M3 (Odds ratio [OR] = 0.103, *p* = 0.046), residual tumor size > 1.5 cm (OR = 0.278, *p* = 0.043), and longer ISP (OR = 0.906, *p* = 0.013)—particularly beyond 31 days (OR = 0.111, *p* = 0.001)—as factors associated with a lower likelihood of CR. Multivariate analysis further confirmed that ISP was the only independent factor correlated with CR (OR = 0.884, *p* = 0.009).

**Conclusion:**

This study highlights that the timely initiation of PBT following surgery, particularly within 31 days, is associated with improved CR in pediatric medulloblastoma. Our findings suggest that a shorter interval between surgery and PBT initiation may be an important factor influencing early treatment response, providing preliminary evidence to support earlier postoperative PBT delivery.

## 1 Introduction

Medulloblastoma is one of the most common malignant brain tumors in children, characterized by its invasive and rapidly growing nature [1]. It typically arises in the posterior fossa of the brain, which contains critical structures such as the cerebellum [2]. Standard treatment consists of maximal safe surgical resection followed by radiation therapy and chemotherapy in most patients [3]. Specific treatments are based on risk stratification, molecular subtype, patient age, and multidisciplinary recommendations. With contemporary protocols, standard‐risk patients achieve 5‐year overall survival (OS) rates of approximately 85% [4], whereas outcomes remain poor in high‐risk patients and very young children, with 5‐year OS below 40% [5] and 45.6% ± 11.7% in children under three years old [6].

Postoperative radiotherapy remains a cornerstone of medulloblastoma management and is consistently associated with improved disease control and survival [7, 8]. Some studies have also suggested that postoperative chemotherapy may provide benefit in selected patient populations, including intensified multimodal regimens for metastatic disease [9, 10]. Patients with medulloblastoma who underwent radiation therapy had significantly longer survival times compared to those who did not receive it [11]. Nevertheless, radiation therapy may also lead to long‐term radiation‐related side effects, including neurological and cognitive deficits, as well as endocrine deficits (which can cause growth problems in children), affecting the survivor's quality of life [12]. Among the available radiotherapy options, proton beam therapy (PBT) has emerged as a leading modality due to its superior precision [13, 14]. PBT delivers high doses of radiation directly to the tumor while sparing surrounding healthy tissues [15, 16], a critical consideration in pediatric patients where long‐term side effects can impair growth and neurological development [17, 18].

Despite the known benefits of PBT, there remain gaps in understanding the factors that influence tumor response following PBT. While PBT has demonstrated high efficacy in improving OS and reducing recurrence in medulloblastoma patients [19], the optimal timing for initiating PBT after surgery, as well as other prognostic factors, remains unclear. Specifically, the interval time between surgery and PBT initiation (ISP) has not been well‐defined, and its impact on clinical outcomes is still under investigation.

This study aims to address these gaps by retrospectively analyzing data from pediatric medulloblastoma patients treated with PBT. Our objective is to explore the relationship between ISP and tumor response, particularly complete response (CR), as well as to identify other clinical factors that may predict treatment success.

## 2 Materials and Methods

### 2.1 Study Design and Patients

**FIGURE 1:**
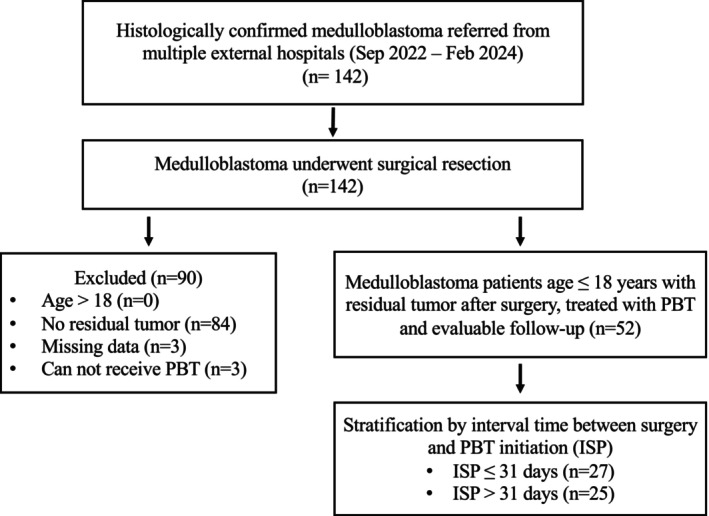
Study flow diagram. A total of 142 pediatric patients with histologically confirmed medulloblastoma referred from external hospitals were screened. After exclusions, 52 patients with postoperative residual tumors who received PBT and had evaluable follow‐up were included. Patients were stratified according to the ISP: ≤ 31 days (*n* = 27) and > 31 days (*n* = 25). PBT, proton beam therapy; ISP, interval time between surgery and PBT initiation.

A retrospective analysis was conducted on 52 pediatric patients with postoperative residual medulloblastoma and treated with PBT between September 2022 and February 2024 (Figure 1). Most patients were referred from multiple external hospitals after initial management, a real‐world referral pattern for specialized PBT. Patients were included if they were younger than 18 years old at the time of diagnosis and had histologically confirmed medulloblastoma in accordance with the 2021 WHO Classification of Tumors of the Central Nervous System. Patient characteristics, treatment details, and other information were obtained from a combination of previous and current medical records.

All data analyzed in this study were collected from Hebei Yizhou Cancer Hospital, Hebei, P.R. China. This study was approved by the Ethics Committee of Hebei Yizhou Cancer Hospital and was conducted in accordance with institutional ethical guidelines. Written informed consent was obtained from the parents or legal guardians of all patients prior to data collection.

The extent of surgical resection was assessed through the surgeon's report from the referring hospital and confirmed by the most recent magnetic resonance imaging (MRI). Tumor staging was based on the Chang staging system, where M0 indicates no evidence of metastatic spread, M1 refers to microscopic tumor cells found in the cerebrospinal fluid (CSF), M2 denotes nodular seeding in the brain beyond the primary site, M3 indicates spinal cord dissemination, and M4 represents metastasis outside the central nervous system.

Tumor classification was based on both histological and molecular assessments. Histological classification was performed using standard microscopic evaluation and classified tumors into subtypes: Desmoplastic/nodular (DN), classic, and large cell/anaplastic (LC/A). Molecular classification involved gene expression profiling and pathway analysis, categorizing tumors into four subgroups: SHH‐activated, Group 3 (G3), Group 4 (G4), and WNT‐activated. Patients were stratified into standard‐risk and high‐risk groups according to established clinical criteria and available pathological information. Standard‐risk patients were defined as being older than 3 years, without metastatic disease (Chang M0), with minimal residual tumor (≤ 1.5 cm^2^), and without unfavorable histologic features. High‐risk patients were classified as those with metastatic disease (Chang M1–M4), significant residual tumor (> 1.5 cm^2^), infant age (≤ 3 years), or unfavorable histology (LC/A). Genetic marker factors (e.g., MYC amplification, TP53 mutation) were incorporated when available.

The primary endpoint of the study was the tumor response following PBT. Tumor response was assessed through MRI by two radiologists and two radiation oncologists and was further classified into four response types: CR, PR, SD, or PD. CR was defined as the absence of detectable tumor, PR as a reduction in tumor size by at least 30%, SD as the absence of significant tumor shrinkage or progression, and PD as the evidence of tumor growth or new lesions during or after treatment.

### 2.2 PBT

All patients included in this study underwent PBT using intensity‐modulated proton therapy (IMPT) to treat pediatric medulloblastoma post‐surgery. The treatment protocol consisted of craniospinal irradiation (CSI) followed by a boost to the posterior fossa or the primary tumor site. IMPT was selected for its superior ability to deliver precise, conformal doses to the tumor while minimizing radiation exposure to surrounding healthy tissues, especially critical in pediatric patients, to reduce the risk of long‐term side effects.

CSI was administered to cover the entire neuraxis, including the brain, spinal cord, and cerebrospinal fluid pathways. The dose and fractionation for CSI were determined based on each patient's clinical risk classification (standard or high) and residual tumor status following surgery. Generally, patients classified as standard‐risk received a CSI dose of 23.4 Gy (RBE) delivered in 1.8 Gy (RBE) fractions. In contrast, high‐risk patients, particularly those with evidence of metastatic disease or large residual tumors, received an escalated CSI dose of up to 36 Gy (RBE) in similar fractionation.

All patients received a posterior fossa or tumor site boost. The boost dose was determined by factors including the residual tumor size and location, as well as the patient's risk classification. Standard‐risk patients received an additional boost dose of 30.6 Gy (RBE) to the posterior fossa, resulting in a total dose of 54 Gy (RBE). High‐risk patients received a total dose of 55.8 Gy (RBE) to the tumor bed. The boost was administered in daily fractions of 1.8 Gy (RBE), ensuring appropriate tumor coverage while limiting radiation to surrounding healthy tissues.

The ISP was defined as the interval time from the completion of surgery to the initiation of PBT. In this study, based on the median ISP of 31 days, patients were categorized into two groups: Those who commenced PBT within 31 days post‐surgery and those who started after 31 days, allowing for the evaluation of ISP's impact on tumor response.

### 2.3 Statistical Analysis

Statistical analyses were performed using SPSS software (version 26.0). The normality of continuous data was evaluated using the Kolmogorov–Smirnov test. Data conforming to a normal distribution were presented as mean ± standard deviation (x̄ ± s), while non‐normally distributed data were expressed as median (M) and interquartile range (P25–P75). Categorical variables were reported as counts and percentages (*n*, %). For categorical variables, differences between groups were assessed using the chi‐square test. When assumptions for the chi‐square test were violated, either Fisher's exact test or the continuity‐corrected chi‐square test was used. As for continuous variables, for normally distributed data with equal variance, an independent samples *t*‐test was applied to compare two groups. The *t*‐test was used when normality was confirmed by the Kolmogorov–Smirnov test and variances were equal. Non‐normally distributed data were compared using non‐parametric tests. Univariate logistic regression was performed to evaluate associations between various clinical factors and the likelihood of achieving a CR as the dependent variable. For the multivariate logistic regression analysis, variables with a *P*‐value < 0.05 in the univariate analysis were entered into the model to assess their independent effects on CR.

A series of sensitivity analyses was conducted to address potential concerns regarding model robustness and the functional form of ISP using R software (version 4.5.2). To mitigate the issue of complete separation in small‐sample data, the primary analyses were re‐estimated using Firth's penalized maximum likelihood logistic regression implemented in the logistf package (version 1.26.1). Second, a generalized additive model (GAM) using penalized spline smoothing was fitted with the mgcv package (version 1.9.4) to examine the assumption of linearity between ISP and CR. Third, the influence of outliers was assessed by calculating Cook's distance to identify influential observations. Models were subsequently refitted after excluding extreme ISP values to evaluate the stability of the estimates. Furthermore, based on the Firth regression model, ISP was dichotomized at the median value (31 days), and bootstrap resampling (1,000 repetitions) was performed using the boot package (version 1.3.32) to further assess the robustness of the ISP effect.

Statistical significance for all tests was established at a *P*‐value < 0.05.

## 3 Results

### 3.1 Patient Characteristics and PBT

A total of 52 pediatric patients with medulloblastoma were included in the study. The median follow‐up period was 12.5 months (5.3–20.3 months). As shown in Table 1, 37 patients (71.2%) were male, and 15 patients (28.8%) were female. The median age at the time of PBT was 6 years, with an age range of 1 to 11 years. Based on the Chang staging system, 34 patients (65.3%) were classified as M0, indicating no evidence of metastatic spread, while 4 patients (7.7%) were M1, 7 patients (13.5%) were M2, and 7 patients (13.5%) were M3, reflecting varying levels of tumor dissemination. 35 patients (67.3%) had a residual tumor size of ≤ 1.5 cm, while 17 patients (32.7%) had a residual tumor size of > 1.5 cm. Additionally, brain dissemination was observed in 5 patients (9.6%), and 6 patients (11.5%) had spinal cord dissemination.

**TABLE 1:** Patient characteristics.

Characteristics	No (%)
**Sex**	
Male	37 (71.2%)
Female	15 (28.8%)
Median age at PBT (range)	6 (1–11)
**Chang staging**	
M0	34 (65.3%)
M1	4 (7.7%)
M2	7 (13.5%)
M3	7 (13.5%)
**Residual tumor size**	
≤ 1.5 cm	35 (67.3%)
> 1.5 cm	17 (32.7%)
**Brain dissemination**	
Yes	5 (9.6%)
No	47 (90.4%)
**Spinal cord dissemination**	
Yes	6 (11.5%)
No	46 (88.5%)
**Histological classification**	
Classic	27 (51.9%)
DN	13 (25%)
LC/A	4 (7.7%)
Not detected	8 (15.4%)
**Molecular classification**	
G3	9 (17.3%)
G3/G4	1 (2.0%)
G4	18 (34.6%)
SHH	15 (28.8%)
WNT	7 (13.5%)
Not detected	2 (3.8%)
**Clinical risk classification**	
High	26 (50%)
Standard	26 (50%)
CSI	28.8 (23.4–39.6)
23.4 Gy (RBE)	24 (46.2%)
> 23.4 Gy (RBE)	28 (53.8%)
Fraction (range)	16 (13–22)
Total dose (RBE) (range)	54 (50.4–55.8)
**Chemotherapy before PBT**	
Yes	12 (23.1%)
No	40 (76.9%)
ISP (range, days)	31 (21–388)
≤ 31 Days	27 (51.9%)
> 31 Days	25 (48.1%)

Abbreviations: DN, desmoplastic/nodular; G3, group 3; G4, group 4; ISP, interval time between surgery and PBT initiation; LC/A, large cell/anaplastic; PBT, proton beam therapy; SHH, sonic hedgehog; WNT, wingless.

Histologically, 27 patients (51.9%) had the classic subtype of medulloblastoma, 13 patients (25%) had the DN subtype, and 4 patients (7.7%) had LC/A medulloblastoma. Histological subtyping was not detected in 8 patients (15.4%). Molecular classification revealed that 9 patients (17.3%) were categorized as G3, 18 patients (34.6%) as G4, 15 patients (28.8%) as SHH‐activated, 1 patient (2.0%) as G3/G4 (composed of both G3 and G4 tumors), and 7 patients (13.5%) as WNT‐activated. It was not detected in 2 patients (3.8%). Patients were further divided into high‐risk (26 patients, 50%) and standard‐risk (26 patients, 50%) categories. Regarding CSI, 24 patients (46.2%) received a CSI dose of 23.4 Gy (RBE), while 28 patients (53.8%) received a higher dose of > 23.4 Gy (RBE). The number of fractions ranged from 13 to 22, with a median of 16 fractions, and the total dose ranged from 50.4 to 55.8 Gy (RBE), with a median of 54 Gy (RBE). 12 patients (23.1%) underwent chemotherapy before PBT, while the remaining 40 patients did not receive chemotherapy. Finally, the ISP was ≤ 31 days for 27 patients (51.9%) and > 31 days for 25 patients (48.1%).

**FIGURE 2:**
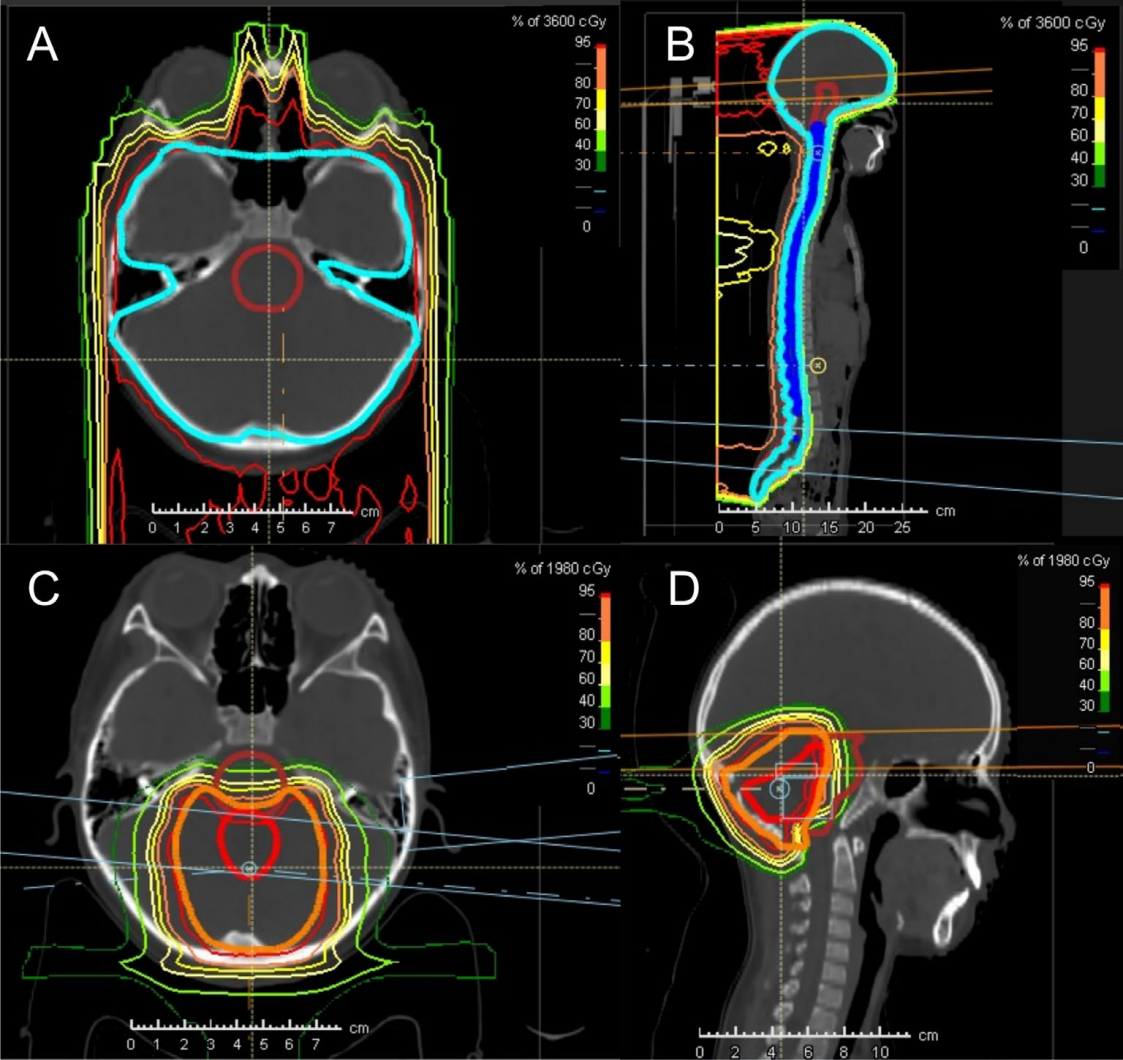
PBT planning for a high‐risk case of medulloblastoma. The figure displays the PBT dose distribution for a medulloblastoma patient, including a CSI dose of 36 Gy (A: Axial panel, B: Sagittal panel), followed by a boost dose of 19.8 Gy to the primary tumor site (C: Axial panel, D: Sagittal panel). The dose color wash and isodose lines illustrate the proton dose delivered, with different colors representing varying dose levels. The planning target volume is outlined in red, while the brainstem as an organ at risk is contoured in deep red. The images demonstrate precise dose conformality to the target while sparing surrounding critical structures, highlighting the advantage of PBT in minimizing radiation exposure to healthy tissues. PBT, proton beam therapy; CSI, craniospinal irradiation.

Figure 2 displays the PBT dose distribution for a high‐risk medulloblastoma patient, including a CSI dose of 36 Gy (Figure 2A,B), followed by a boost dose of 19.8 Gy to the primary tumor site (Figure 2C,D). The dose distribution demonstrates precise dose conformality to the target while sparing surrounding critical structures, such as the brainstem, highlighting the advantage of PBT in minimizing radiation exposure to healthy tissues.

### 3.2 Tumor Response Post‐PBT and the ISP

**FIGURE 3:**
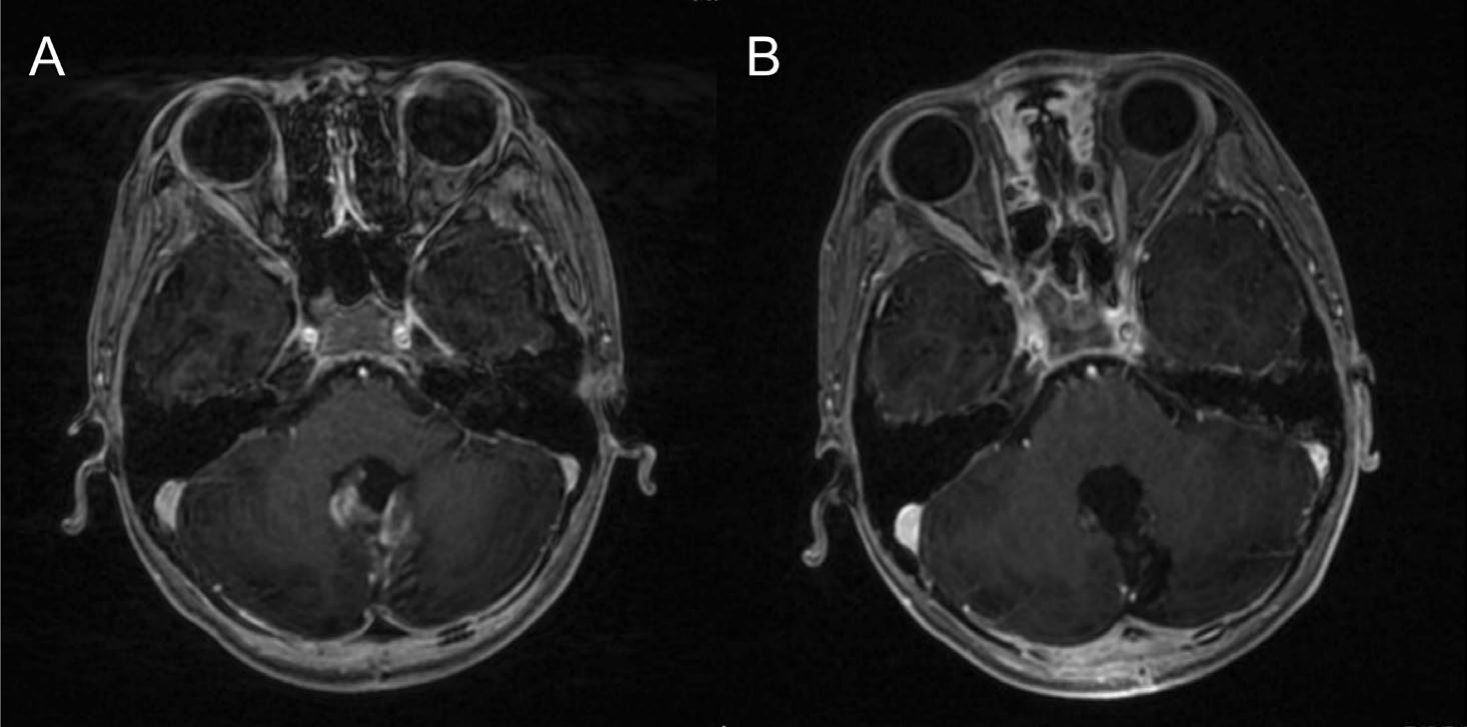
MRI images of a medulloblastoma patient before and after PBT. (A) Axial T1‐weighted MRI with contrast post‐surgery shows a residual medulloblastoma tumor in the posterior fossa region before PBT. The residual tumor appears as an enhanced mass adjacent to the brainstem. (B) Axial T1‐weighted MRI with contrast after PBT demonstrates a significant reduction in tumor size, with no visible enhancement in the treated area, indicating a CR to the therapy. MRI, magnetic resonance imaging; PBT, proton beam therapy; CR, complete response.

**FIGURE 4:**
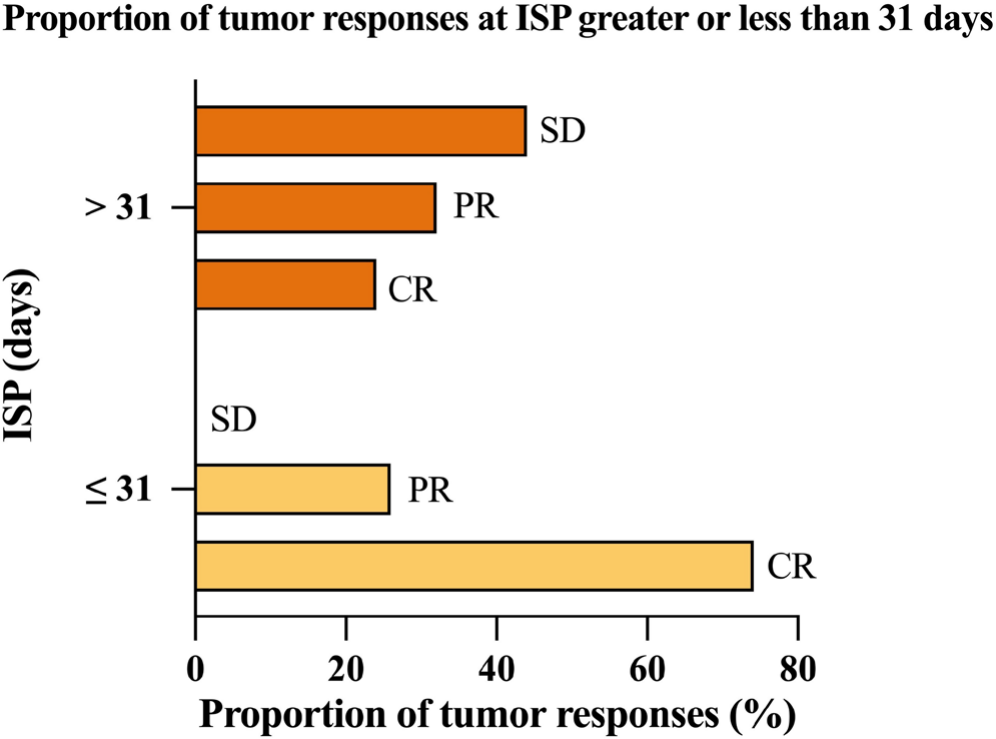
Proportion of tumor responses at ISP greater than or less than 31 days. This figure illustrates the distribution of tumor response types based on the ISP. The data is divided into two groups: Patients with ISP ≤ 31 days and those with ISP > 31 days. CR was a significantly higher proportion in the ISP ≤ 31 days group compared to the ISP > 31 days group. SD showed higher representation in the ISP > 31 days group than in the ISP ≤ 31 days group. ISP, interval between surgery and proton beam therapy; CR, complete response; PR, partial response; SD, stable disease.

Treatment response was assessable in all 52 patients, with 26 (50.00%) patients in CR, 15 (28.85%) patients in PR, and 11 (21.15%) patients with SD. Figure 3 shows a case of residual medulloblastoma in the posterior fossa region after surgery. The MRI after PBT (Figure 3B) demonstrates a significant reduction in tumor size, with no enhancement observed in the treated area, indicating a CR of the tumor. Figure 4 illustrates the distribution of tumor response types based on the ISP. The data were divided into two groups: Patients with ISP ≤ 31 days and those with ISP > 31 days. CR was a higher proportion in the ISP ≤ 31 days group (74.07%) compared to the ISP > 31 days group (24.00%). SD showed higher representation in the ISP > 31 days group (44.00%) than in the ISP ≤ 31 days group (no cases of SD were observed). Comparisons showed that CR was more likely to occur when the ISP duration was short, and the proportion of SD became higher when the ISP duration became longer.

**FIGURE 5:**
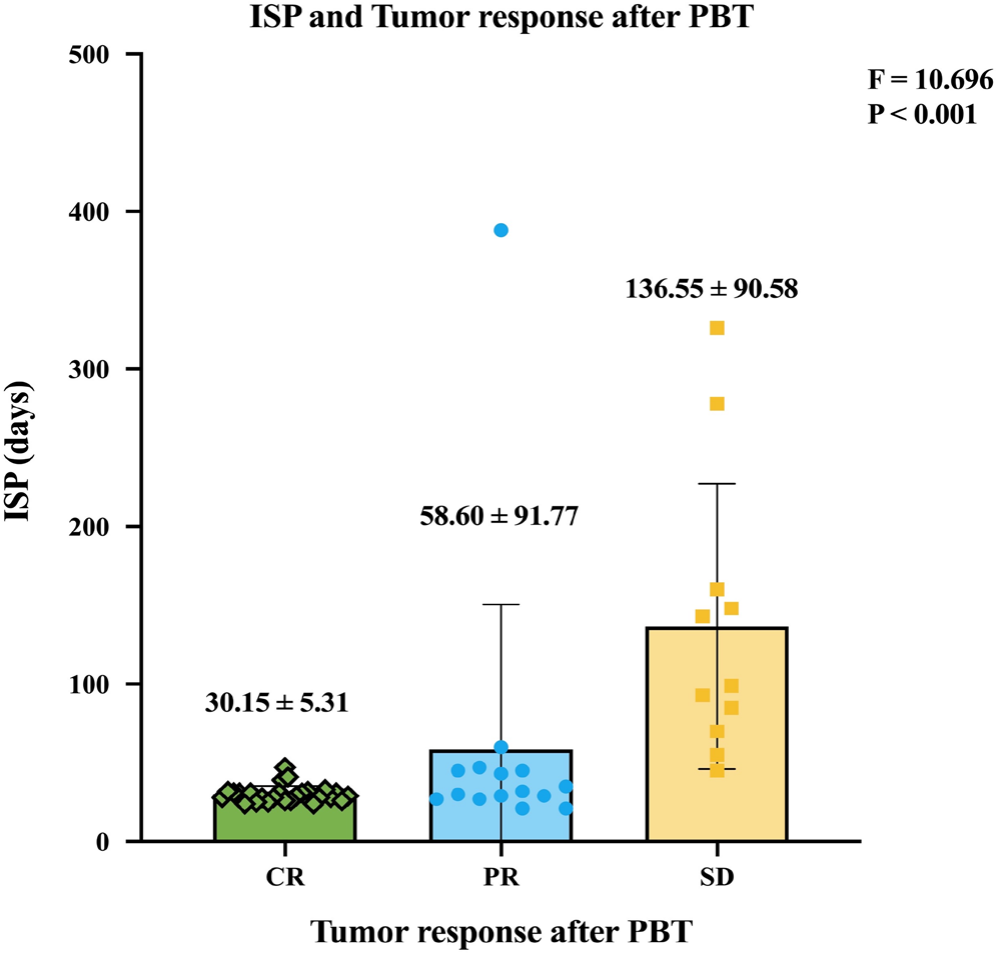
ISP and tumor responses after PBT. The bar graph illustrates the mean interval time (± standard deviation) between surgery and PBT for 52 patients categorized by tumor response: CR, PR, and SD. The mean interval times were 30.15 ± 5.31 days for CR, 58.60 ± 91.77 days for PR, and 136.55 ± 90.58 days for SD. Statistical analysis revealed a difference among the groups (F = 10.696, *p* < 0.001), indicating that shorter interval times are associated with better tumor response outcomes. ISP, interval time between surgery and PBT initiation; PBT, proton beam therapy; CR, complete response; PR, partial response; SD, stable disease.

Figure 5 further illustrates the relationship between tumor response and the ISP. The mean interval time for patients achieving CR was shorter (30.15 ± 5.31 days) compared to those with PR (58.60 ± 91.77 days) and SD (136.55 ± 90.58 days). Statistical analysis using analysis of variance (ANOVA) revealed a significant difference in ISP between the tumor response groups, with an F‐value of 10.696 and a *P*‐value of < 0.001, indicating that shorter ISP was strongly associated with improved tumor response (CR).

### 3.3 Analysis of Impact Factors of CR and Non‐CR

Various clinical factors influencing tumor response were analyzed in patients undergoing PBT (Table 2). Sex did not show a statistically significant difference (*p* = 0.126). Age also did not demonstrate a different impact on tumor response (*p* = 0.245), with mean ages of 6.50 and 5.50 years for the CR and non‐CR groups, respectively. The Chang staging presented a trend suggesting that patients with the earlier stage (M0) were more likely to achieve CR. However, this trend was not statistically significant (*p* = 0.081). In contrast, residual tumor size showed a significant association. Patients with smaller residual tumor sizes (≤ 1.5 cm) had higher CR rates (80.77%) compared to those with larger tumors (> 1.5 cm) (*p* = 0.039). Moreover, brain dissemination significantly affected tumor response (*p* = 0.019). All patients without brain dissemination achieved CR, whereas those with brain dissemination were more likely to have a non‐CR outcome (19.23%). Although spinal cord dissemination did not reach statistical significance (*p* = 0.083), it was more frequent in the non‐CR group.

**TABLE 2:** Clinical factors according to the tumor response.

Index	Tumor response		X^2^/t	*P*‐value
	CR (*n* = 26)	Non‐CR (*n* = 26)		
**Sex**			2.342	0.126
Male	21 (80.77%)	16 (61.54%)		
Female	5 (19.23%)	10 (38.46%)		
Age	6.50 ± 2.80	5.50 ± 3.30	1.177	0.245
**Chang staging**			6.739	0.081
M0	21 (80.77%)	13 (50.00%)		
M1	2 (7.69%)	2 (7.69%)		
M2	2 (7.69%)	5 (19.23%)		
M3	1 (3.85%)	6 (23.08%)		
**Residual tumor size**			4.282	0.039
≤ 1.5 cm	21 (80.77%)	14 (53.85%)		
> 1.5 cm	5 (19.23%)	12 (46.15%)		
**Brain dissemination**			5.532	0.019
Yes	0 (0.00%)	5 (19.23%)		
No	26 (100.00%)	21 (80.77%)		
**Spinal cord dissemination**			3.014	0.083
Yes	1 (3.85%)	5 (19.23%)		
No	25 (96.15%)	21 (80.77%)		
**Histological classification**			0.114	0.990
Classic	14 (53.85%)	13 (50.00%)		
DN	6 (23.08%)	7 (26.92%)		
LC/A	2 (7.69%)	2 (7.69%)		
Not detected	4 (15.38%)	4 (15.38%)		
**Molecular classification**			5.638	0.343
G3	5 (19.23%)	4 (15.38%)		
G3/G4	0	1 (3.85%)		
G4	7 (26.92%)	11 (42.31%)		
SHH	7 (26.92%)	8 (30.77%)		
WNT	6 (23.08%)	1 (3.85%)		
Not detected	1 (3.85%)	1 (3.85%)		
**Clinical risk classification**			1.231	0.267
High	11 (42.31%)	15 (57.69%)		
Standard	15 (57.69%)	11 (42.31%)		
CSI dose			2.786	0.095
23.4 Gy	15 (57.69%)	9 (34.62%)		
> 23.4 Gy	11 (42.31%)	17 (65.38%)		
Total dose	54.81 ± 1.36	54.88 ± 0.94	−0.208	0.836
**Chemotherapy before PBT**			15.6	< 0.001
Yes	0 (0.00%)	12 (46.15%)		
No	26 (100.00%)	14 (53.85%)		
ISP			13.019	< 0.001
≤ 31	20 (76.92%)	7 (26.92%)		
> 31	6 (23.08%)	19 (73.08%)		

Abbreviations: CR, complete response; CSI, craniospinal irradiation; DN, desmoplastic/nodular; G3, group 3; G4, group 4; ISP, interval time between surgery and PBT initiation; LC/A, large cell/anaplastic; PBT, proton beam therapy; SHH, sonic hedgehog; WNT, wingless.

Histological classification, molecular classification, clinical risk classification, CSI dose, and total radiation dose all showed no significant correlation with tumor response (*p* = 0.990, 0.343, 0.267, 0.095, and 0.836, respectively). These findings suggest that these factors may not play major roles in predicting CR outcomes. However, the pre‐PBT chemotherapy and the ISP showed different impacts (both *p* < 0.001). Among the patients with CR, all 26 (100%) patients had not received chemotherapy, whereas 12 patients who had received chemotherapy (46.15%) presented with non‐CR on PBT. Patients who began PBT within 31 days post‐surgery had a higher CR rate (76.92%) compared to those who started PBT after more than 31 days (23.08%), emphasizing the importance of PBT initiation timing in achieving favorable outcomes.

This analysis highlights that residual tumor size, brain dissemination, chemotherapy before PBT, and the interval from surgery to PBT are critical determinants in predicting CR outcomes in patients receiving PBT.

### 3.4 Univariate and Multivariate Logistic Regression Analysis of CR

To provide further insights into the strength and direction of the association between each variable and the likelihood of achieving CR, factors with a *p*‐value < 0.1 in the group analysis described in Table 2 were included and analyzed in the univariate logistic regression analysis. However, the factors of brain dissemination and chemotherapy before PBT were excluded due to a lack of variability (no CR cases in the brain dissemination group and the chemotherapy before PBT group), making it impossible for logistic regression to estimate the odds ratio (OR) for these factors accurately, as logistic regression requires some outcome variation within each group to calculate associations. Table 3 presents the univariate logistic regression analysis of various factors influencing CR as the dependent variable. ORs with 95% confidence intervals (CIs) were calculated to quantify the likelihood of achieving CR based on these factors. According to the results of the analysis, factors *P*‐value < 0.05, Chang staging M3, residual tumor size > 1.5 cm, longer ISP, and the ISP of > 31 days, were associated with a lower likelihood of achieving CR. Of these, ISP showed significance with a *P*‐value of 0.013. The *β* value of −0.099 and OR of 0.906 suggest that increasing the interval time (as a continuous variable) reduces the likelihood of achieving CR. The probability of getting a CR decreases to 0.906 for each additional day of the interval. For each day of delay in initiating PBT, the probability of CR decreased to 0.906. In addition, ISP (≤ 31 vs. > 31 days) was a different variable with a *P*‐value of 0.001. The *β* value of −2.203 suggests a reduction in the odds of CR when the interval time between surgery and PBT was greater than 31 days. The OR was 0.111, indicating that delaying PBT for more than 31 days reduced the likelihood of obtaining a CR to 0.111.

**TABLE 3:** Univariate logistic regression analysis with CR as the dependent variable.

Items	β	S_b_	Wald^2^	P‐value	ORs	95% CI Lower limit	Upper limit
Chang staging							
M0	Reference						
M1	−0.48	1.06	0.205	0.651	0.619	0.077	4.947
M2	−1.396	0.908	2.363	0.124	0.248	0.042	1.468
M3	−2.271	1.136	3.995	0.046	0.103	0.011	0.957
Residual tumor size > 1.5 cm (No vs. Yes)	−1.281	0.634	4.078	0.043	0.278	0.08	0.963
Spinal cord dissemination (No vs. Yes)	−1.281	0.871	2.164	0.141	0.278	0.05	1.531
CSI dose	−0.087	0.046	3.618	0.057	0.917	0.838	1.003
ISP	−0.099	0.04	6.184	0.013	0.906	0.837	0.979
ISP (≤ 31 vs. > 31)	−2.203	0.642	11.77	0.001	0.111	0.031	0.389

Abbreviations: CI, confidence interval; CR, complete response; CSI, craniospinal irradiation; ISP, interval time between surgery and PBT initiation; ORs, odds ratios.

For the multivariate logistic regression analysis (Table 4), variables that were different in the univariate logistic regression analysis (*p* < 0.05) were further entered into the model to assess their independent effects on CR. And it was analyzed which factor among the above variables with differences had the strongest correlation with CR. Multivariate logistic regression analysis revealed that, after adjusting for other factors, only the ISP remained statistically different in predicting CR (*p* = 0.009). The β value of −0.123 indicated that as the interval time between surgery and PBT increased, the likelihood of achieving CR decreased. Each additional day of delay reduced the likelihood of achieving CR by 11.6% (OR = 0.884, 95% CI = 0.806–0.97). Other factors, such as Chang staging and residual tumor size, were not statistically different in this multivariate analysis, even though they showed some significance in the univariate analysis. This suggested that their impacts were less pronounced when considering the other variables together. The interval time was the factor with the greatest correlation of influence on the occurrence of CR, and it affected the occurrence of CR independently.

**TABLE 4:** Multivariate logistic regression analysis with CR as the independent variable.

Items	β	S_b_	Wald^2^	*P*‐value	ORs	95% CI	
						Lower limit	Upper limit
Chang staging							
M0	Reference						
M1	1.255	1.678	0.56	0.454	3.51	0.131	94.147
M2	−1.099	1.161	0.897	0.344	0.333	0.034	3.242
M3	−1.748	1.5	1.359	0.244	0.174	0.009	3.29
Residual tumor size > 1.5 cm (No vs. Yes)	−1.244	1.039	1.434	0.231	0.288	0.038	2.208
ISP	−0.123	0.047	6.852	0.009	0.884	0.806	0.97

Abbreviations: CI, confidence interval; CR, complete response; ISP, interval time between surgery and PBT initiation; ORs, odds ratios.

### 3.5 Sensitivity Analyses Assessing Model Robustness

**FIGURE 6:**
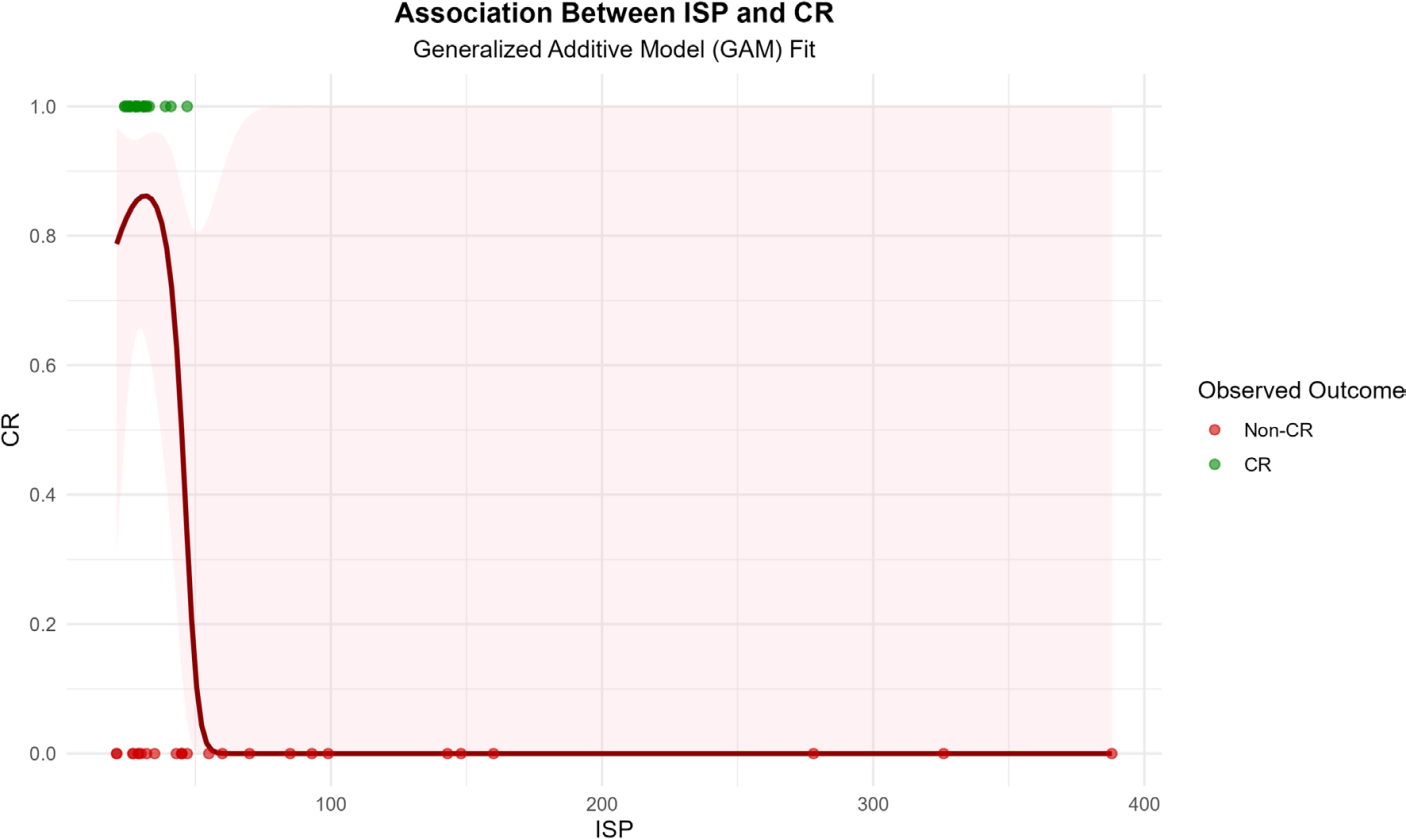
Association between ISP and CR modeled by a GAM. The figure illustrates the nonlinear relationship between ISP and the probability of achieving CR using a GAM. Individual patient outcomes are shown as jittered points, with green dots indicating CR and red dots indicating non‐CR. A rapid decrease in CR probability is observed when ISP is less than 100 days, followed by a plateau at lower levels. Although a nonlinear pattern is visually suggested, formal statistical testing did not show a significant departure from linearity (*p* = 0.126) (Table 5), supporting the use of ISP as a continuous linear variable in the primary analysis. ISP, interval time between surgery and PBT initiation; CR, complete response; GAM, generalized additive model.

To address potential concerns regarding model robustness and the functional form of ISP, sensitivity analyses were performed (Table 5). First, the primary analysis was re‐estimated using Firth's penalized maximum likelihood logistic regression to account for complete separation in the small‐sample dataset. The results showed that for each 1‐day increase in ISP, the odds of achieving CR decreased by 8.9% (OR = 0.911, 95% CI: 0.828–0.975, *p* < 0.001). The GAM revealed a nonlinear association between ISP and the probability of CR (Figure 6). When ISP was less than 100 days, the predicted probability of CR declined rapidly with increasing ISP, whereas it remained relatively stable at a low level when ISP exceeded 100 days. Although the fitted curve visually suggested a nonlinear trend, formal statistical testing did not provide sufficient evidence to reject the null hypothesis of linearity (*p* = 0.126). Therefore, treating ISP as a continuous linear variable in the primary analysis was considered appropriate and statistically justified.

**TABLE 5:** Sensitivity analysis results for model robustness.

Analysis scenario	Sample size	ISP effect	95% CI or visual	*P*‐value
Firth Penalized Regression (Primary)	52	OR = 0.911	0.828–0.975	< 0.001
Cook's Distance Diagnostics Excluding ISP > 138.6 days	46	OR = 0.911	0.828–0.973	0.0011
ISP Median Dichotomy (31 days)	52	OR = 0.109	0.023–0.399	< 0.001
Bootstrap (Firth‐based)	52	Median OR = 0.907	0.805–0.988	N/A

Abbreviations: CI, confidence interval; CR, complete response; CSI, craniospinal irradiation; ISP, interval time between surgery and PBT initiation; ORs, odd ratios.

**FIGURE 7:**
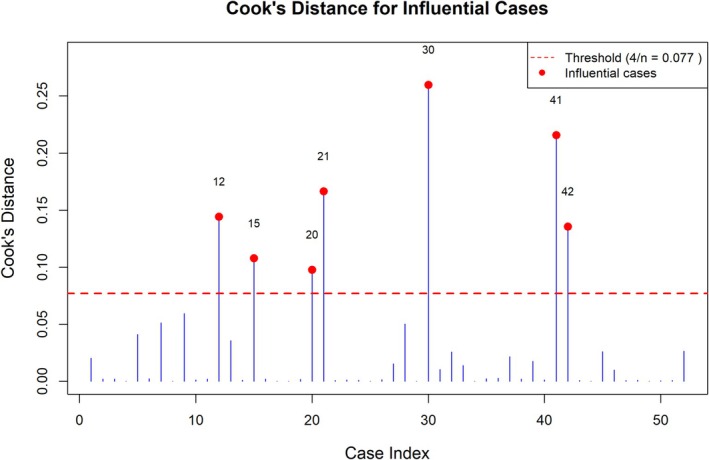
Cook's distance for influential observations. Cook's distance was calculated to identify influential cases in the logistic regression model. The dashed red horizontal line indicates the commonly used threshold (4/*n* = 0.077), where n represents the sample size. Observations exceeding this threshold were considered potentially influential and are highlighted in red. The diagnostics identified seven influential observations (indices 12, 15, 20, 21, 30, 41, and 42).

Cook's distance diagnostics identified seven influential observations (Figure 7). After excluding six extreme cases with ISP > 138.6 days, the results remained unchanged (OR = 0.911, *p* = 0.0011), indicating that the findings were not driven by a small number of outliers. In addition, based on the Firth regression model, ISP was dichotomized at the median value (31 days), and patients with ISP > 31 days showed an 89.1% reduction in the odds of achieving CR (OR = 0.109, *p* < 0.001), supporting 31 days as a clinically meaningful cutoff point. Furthermore, bootstrap resampling was performed and supported the robustness of the ISP effect (median OR = 0.907, 95% CI: 0.805–0.988).

## 4 Discussion

### 4.1 Advancements in Medulloblastoma Treatment: PBT

The management of pediatric medulloblastoma has evolved significantly, with multimodal approaches combining surgery, chemotherapy, and advanced radiation techniques becoming recommended [20]. Despite improvements in survival rates, medulloblastoma remains a complex challenge, particularly in high‐risk groups, due to its aggressive behavior and potential for dissemination [21]. PBT, with its ability to deliver targeted doses while minimizing damage to surrounding healthy tissue, represents a crucial advancement, especially for pediatric patients who are more susceptible to long‐term radiation‐related side effects [22]. However, optimizing the timing of PBT initiation post‐surgery to maximize its effectiveness remains an area of investigation.

### 4.2 Postoperative Radiotherapy Vs. Chemotherapy

In fact, the timing of postoperative radiotherapy is one of the most important factors affecting treatment outcome and patient prognosis. For head and neck cancer, the American National Comprehensive Cancer Network (NCCN) recommends that planned radiotherapy begin within 42 days after surgery, while the Dutch Head and Neck Society recommends 30 days post‐surgery [23]. A retrospective study of 41,291 head and neck cancer patients demonstrated that non‐compliance with NCCN guidelines (starting postoperative radiotherapy within 6 weeks of surgery) was associated with decreased survival [24]. Another study on maxillary sinus cancer found that initiating postoperative radiotherapy within 4 weeks significantly prolonged OS, with radiotherapy started within 3–4 weeks showing the most marked improvement in prognosis [25].

In medulloblastoma, prioritizing postoperative chemotherapy may delay the initiation of radiotherapy. A report from the German Society of Pediatric Hematology and Oncology revealed that compared to maintenance chemotherapy after immediate postoperative radiotherapy for medulloblastomas, the postoperative neoadjuvant chemotherapy before radiation therapy resulted in increased bone marrow toxicity, and delayed radiotherapy may negatively affect survival outcomes. High‐quality radiotherapy might be a major influence on overall outcome [8]. After a median 10‐year follow‐up, patients who received chemotherapy after radiotherapy had higher OS rates compared to those who received chemotherapy before radiotherapy, for both Chang staging M0 (10‐year OS 91% and 62%, *p* = 0.001) and M1 patients (10‐year OS 70% and 34%, *p* = 0.020) [7]. In terms of photon radiotherapy timing for medulloblastoma, the current Children's Oncology Group ACNS0332 trial for high‐risk medulloblastoma recommended starting RT within 31 days of surgery, while St Jude's SJMB12 trial specified a 36‐day interval post‐surgery [26]. Compared to patients who started radiotherapy later, those who received radiotherapy within 25 days after resection showed a higher 3‐year OS rate (81.5% vs. 59.5%; *p* = 0.11) and a higher 3‐year disease‐free survival rate (74.1% vs. 46.0%; *p* = 0.03). The interval between surgery and radiotherapy was an important prognostic factor for disease‐free survival [27]. All the above findings indicate that initiating photon radiotherapy immediately after surgery, rather than chemotherapy, is crucial. However, no studies to date have clearly identified the optimal timing for initiating PBT. The results of this study further support the consensus of initiating PBT soon after surgery rather than prioritizing chemotherapy. It should be noted that the administration of chemotherapy before radiotherapy in some patients in our cohort reflects real‐world referral patterns rather than an institutional treatment preference. Most patients in this study were referred from external hospitals. Consequently, initial treatment strategies—including whether to initiate adjuvant chemotherapy before radiotherapy—were determined by the referring institutions based on their clinical judgment and patient status. In addition, practical factors such as referral timing, scheduling availability, and family‐related logistics influenced patients' access to PBT, which may have further contributed to delays in radiotherapy initiation. These constraints explain why a subset of patients received chemotherapy prior to PBT in our cohort. In this study, chemotherapy before PBT impacted CR outcomes, with a *p*‐value less than 0.001 (Table 2). Among the CR group, all 26 patients (100%) had not received chemotherapy before PBT, whereas nearly half (46.15%) of the patients who had undergone chemotherapy prior to PBT did not achieve CR. Although it was not possible for us to further analyze the impact of chemotherapy using univariate regression analysis, it was not the key focus of this study. These findings have suggested that chemotherapy delayed the initiation of PBT to some extent, which may have negatively affected the CR rates.

### 4.3 Balance of Surgery and Radiotherapy for Medulloblastoma

Notably, medulloblastoma surgery poses significant challenges, especially when tumors are detected late due to severe neurological symptoms, necessitating immediate intervention [28, 29]. In such urgent cases, there may not be access to an experienced neurosurgeon, increasing the risk of neurological deficits, hydrocephalus, tumor dissemination, and metastasis during the procedure [30, 31]. Given these risks, partial resection that avoids major neurological damage can be a strategic option, particularly if PBT can be proven to achieve a CR for residual disease. By minimizing surgical complications, the patient can recover quickly, allowing for the timely initiation of PBT, which is crucial for maximizing its effectiveness and improving prognosis [32]. Thus, a balanced approach that prioritizes early PBT while avoiding extensive, high‐risk surgery may offer better long‐term outcomes.

In 2000, Jenkin et al. conducted a retrospective study of 173 cases, reporting 5‐year OS rates by extent of resection: 63% for gross total resection (GTR), 50% for near‐total resection (90%–99%), 41% for partial resection (50%–89%), and 17% for less than 50% partial resection. GTR significantly improved OS compared to non‐total resection groups (*p* = 0.002). However, when standard radiotherapy was administered post‐resection, GTR did not emerge as a significant prognostic factor. Consequently, if achieving GTR holds a high risk of complications, it may be more prudent to limit the extent of resection and ensure that radiotherapy can proceed without interruption due to postoperative complications [33]. In a retrospective study of 203 cases by Akyüz et al., it was reported that the extent of surgical resection did not significantly affect the survival time [34]. When there is a high risk of neurological symptoms due to surgery, it is important to avoid forced total resection and instead proceed promptly with postoperative radiotherapy. In a multi‐institutional collaborative study by Thompson, Eric M et al. in 2016 involving 787 cases, the effect of GTR as a prognostic factor was significantly diminished when molecular subtype classification was considered in the multivariate analysis. GTR prolonged progression‐free survival (PFS) compared to resection with ≥ 1.5 cm^2^ of tumor remaining (hazard ratio [HR] 1.45, 95% CI 1.07–1.96, *p* = 0.16); however, there was no OS benefit (HR 1.23, 0.87–1.72, *p* = 0.24). Additionally, there was no PFS or OS benefit for GTR compared with resection, leaving < 1.5 cm^2^ of tumor remaining (HR 1.05, 0.71–1.53, *p* = 0.8158 for PFS and HR 1.14, 0.75–1.72, *p* = 0.55 for OS). In WNT, SHH, and group 3 subtypes, a greater extent of resection showed no benefit on survival. This supported the established guideline of avoiding high‐risk surgery for small residual portions of medulloblastoma when neurological morbidity was anticipated [35]. Our study demonstrated that residual tumor size > 1.5 cm was significantly associated with a lower likelihood of achieving a CR compared to smaller residual tumors (*p* = 0.043 in univariate analysis). However, we are only suggesting that when it is within safe parameters (without the high risk of nerve impairment and other complications), the residual tumor size should be surgically limited to less than 1.5 cm, and complete resection does not need to be mandatory. This is because, while this association was significant in univariate analysis, it did not remain different in multivariate analysis, indicating that residual tumor size alone did not independently impact CR outcomes when accounting for other factors, such as the ISP. This finding suggested that even if the residual tumor was > 1.5 cm, immediate and timely PBT still had a high likelihood of achieving CR. It is also worth noting that our study evaluated only the short‐term tumor response following PBT. We did not assess the correlation between residual tumor size and long‐term survival, such as OS and PFS, which will be further addressed in future research.

### 4.4 ISP And Other Clinical Factors

In our study, we observed that initiating PBT within 31 days of surgery improved the likelihood of achieving a CR, a timing that is broadly consistent with previous findings on photon radiotherapy. This finding also underscores the critical role of ISP in influencing treatment outcomes. Several factors may explain why shorter ISP correlates with higher CR rates. First, early initiation of PBT may inhibit tumor regrowth and prevent the spread of microscopic disease, which is more likely to occur in the postoperative period when the patient is most vulnerable [36]. Second, shorter ISPs may contribute to maintaining the surgical bed in an optimal state for radiation, enhancing the tumor's radiosensitivity [37]. This observation aligns with broader oncology research, suggesting that minimizing time between surgical intervention and radiotherapy generally improves tumor control rates. However, previous studies have often focused on the overall impact of PBT on medulloblastoma outcomes without detailing the influence of ISP [38, 39]. The results specific to medulloblastoma and PBT have not been thoroughly explored in existing literature, highlighting the novelty and clinical relevance of our findings.

Beyond ISP, our analysis identified other clinical factors such as tumor size, Chang staging, and brain dissemination that are traditionally linked to treatment outcomes. While these factors appeared different in the univariate analysis, the multivariate analysis revealed ISP as the only independent predictor of CR. This indicates that when PBT is initiated within an optimal timeframe, the influence of other variables, such as residual tumor size and metastatic staging, may be minimized. These findings suggest that early intervention with PBT may serve as a compensatory factor that enhances treatment efficacy, even in cases with initially unfavorable prognostic indicators.

Despite these significant findings, the limitations of our study must be considered. One major limitation is the relatively short follow‐up period. While the median follow‐up of 12.5 months allowed for an initial assessment of treatment response, a longer period is necessary to fully evaluate the durability of CR and overall survival rates [40]. A longer follow‐up could provide more robust data on recurrence rates and late radiation‐related effects, offering a comprehensive understanding of the long‐term efficacy of early PBT initiation. Additionally, the relatively small, single‐center cohort may limit the broader applicability of our results. Given the retrospective design and limited cohort size, all statistical analyses should be considered exploratory in nature. No correction for multiple testing was applied; therefore, the results should be interpreted with caution and primarily regarded as hypothesis‐generating rather than confirmatory. The ISP cutoff of 31 days, while statistically validated in our cohort, may need further refinement when applied across diverse populations and clinical settings. Future studies should aim to replicate these findings through multicenter trials that can validate the optimal ISP and investigate the biological underpinnings of these effects. It should also be acknowledged that contemporary risk stratification for medulloblastoma integrates clinical, pathological, and molecular features. However, comprehensive gene biomarker profiling was not available for all patients in this retrospective cohort. Therefore, risk classification in our study relied primarily on clinical and radiologic factors. Future studies incorporating systematic molecular subgrouping are warranted to further refine risk stratification and to better elucidate its interaction with treatment timing and response to PBT.

## 5 Conclusion

Our study provides preliminary evidence supporting the clinical importance of early initiation of PBT in improving early treatment response in pediatric medulloblastoma. Shortening the interval between surgery and PBT initiation, especially to within 31 days, was associated with a higher rate of CR in this retrospective cohort. Longer follow‐up and larger, prospective studies are needed to determine whether early PBT initiation translates into sustained benefits in PFS or OS.

## Author Contributions

Z.S.: Wwriting – original draft, data curation. Z.H.: Fformal analysis, methodology, software, writing – review and editing. W.H.: Resources, Data curation, Methodology. Y.Z.: Resources, Data curation. W.Z.: Resources, Data curation. S.W.: Rresources, data curation. J.L.: Rresources, data curation. C.J.: Rresources, data curation. Z.W.: Rresources, investigation, software. W.W.: Rresources, investigation, software. S.Z.: Iinvestigation, resources. H.S.: Mmethodology, conceptualization. S.S. (Q.X.): Cconceptualization, supervision, methodology, validation, project administration, writing – review and editing.

## Ethics Statement

All procedures performed in the study and involving human participants were performed in accordance with the ethical standards of the institutional and/or national research committee and with the principles of the 1964 Declaration of Helsinki and its later amendments or comparable ethical standards. This retrospective analysis was approved by the Institutional Review Board of Hebei Yizhou Cancer Hospital, Hebei, P.R. China. Informed consent was obtained from all individual participants or their guardians included in the study.

## Consent

Written informed consents were obtained from the patients or their guardians for publication of this study and accompanying images.

## Conflicts of Interest

The authors declare no conflicts of interest.

## Data Availability

The data that support the findings of this study are available on request from the corresponding author. The data are not publicly available due to privacy or ethical restrictions.
